# Comparing Frequency of Listener Responses Between Adolescents with and Without ASD During Conversation

**DOI:** 10.1007/s10803-021-04996-9

**Published:** 2021-04-10

**Authors:** Holly Matthewman, Emily Zane, Ruth Grossman

**Affiliations:** 1 University of Bath, Claverton Down, Bath, BA2 7AY UK; 2 Present Address: James Madison University, 800 S Main St, Harrisonburg, VA 22807 USA; 3 FACE Lab at Emerson College, 120 Boylston Street, Boston, MA 02116 USA

**Keywords:** Autism spectrum disorder, Backchanneling, Communication, Conversation, Eye contact, Gaze, Listener feedback

## Abstract

In conversation, the listener plays an active role in conversation success, specifically by providing *listener feedback* which signals comprehension and interest. Previous work has shown that frequency of feedback positively correlates with conversation success. Because individuals with ASD are known to struggle with various conversational skills, e.g., turn-taking and commenting, this study examines their use of listener feedback by comparing the frequency of feedback produced by 20 adolescents with ASD and 23 neurotypical (NT) adolescents. We coded verbal and nonverbal listener feedback during the time when participants were listening in a semi-structured interview with a research assistant. Results show that ASD participants produced significantly fewer instances of listener feedback than NT adolescents, which likely contributes to difficulties with social interactions.

## Introduction

It is well established that individuals with autism spectrum disorder (ASD) have difficulties with reciprocal social interactions (Lord et al., 2012). Previous research indicates differences in many aspects of speaker behavior, including turn-taking, question-asking, comment-making, and topic-shifting (Arie et al., 2008; Jones & Schwartz, 2009; Koegel et al., 2014). In general, research suggests that individuals with ASD are less likely than neurotypical (NT) peers to initiate conversation in the first place (Jones & Schwartz, 2009), and when they do, they tend to focus on topics of interest to them, use repetitive language, and have difficulty switching topics or asking questions in order to accommodate their conversation partner (Eales, 1993; Carpenter & Tomasello, 2000). They are also less likely to comment on information provided by their communication partner (Jones & Schwartz, 2009). This research has also shown that individuals with ASD attend less to cues from their partner, and this results in them appearing disinterested in the conversation (Klinger & Williams, 2009). Relatedly, NT conversation partners often report that conversation with ASD individuals is awkward and that they perceive the individual with ASD to be boring, rude, disinterested or inappropriate (Brinton & Fujiki, 1989; Marans et al., 2005).

Collectively, these data show that individuals with ASD often do not demonstrate normative conversational behavior as a *speaker*, but very little research has focused on *listener* behaviors in ASD, including the amount and quality of listener feedback they provide during reciprocal social interactions.

### Listener Feedback

Successful conversation depends on the listener showing they are paying attention and following what the speaker is saying (Tolins & Tree, 2014). This is achieved by an active display of attention, interest, understanding, and willingness to let the speaker continue (Ward et al., 2007). Therefore, in dyadic face-to-face conversations the listener does not typically remain mute or motionless, but instead frequently provides verbal and nonverbal signals that help regulate the interaction and provide key semantic and emotional feedback (Jonsdottir et al., 2007).

These verbal and nonverbal signals are referred to as listener feedback and include head nods and shakes, sentence completions, brief restatements and clarification requests (Duncan, 1974; Krauss et al., 1977). Studies have also found that listeners provide feedback in the form of facial feedback (smiles, grimaces, and raised eyebrows), gaze shifts, and laughter (Bertrand et al., 2007; Jonsdottir et al., 2007; Bavelas & Gerwing, 2011). A further important type of listener feedback is short utterances such as ‘m-hm’ or ‘uh-huh’ known as ‘backchanneling’. This term reflects its role within the two channels of communication operating simultaneously in a conversation; the speaker owns the front channel, directing the speech flow and the listener makes minimal, non-interruptive responses, forming the backchannel (Yngve, 1970).

Listener feedback does not add content to the interaction but instead shows that the speaker is being attended to and understood (Schröder et al., 2006). Such feedback, therefore, does not signal an attempt by the listener to take a speaker turn, instead, it serves the purpose of inviting the speaker to continue (Ward & Tsukahara, 2000). Listener feedback is brief so that it does not disrupt the speaker’s turn; feedback either occurs during short pauses between speaker phrases or it overlaps very quickly with the speaker’s words (Goodwin, 1986; Mueller, 1996). In fact, backchanneling brevity specifically has been used as a characteristic that distinguishes it from other conversational behaviors, like commenting (Koiso et al., 1995).

Speakers depend on feedback in order to understand the effect of their words on the listener’s mental state and their thoughts and feelings about what is being said (Schröder et al., 2006). When listeners do not provide feedback, they can appear uninterested or even discourteous to the speaker (Ward et al., 2007). A study by Bavelas et al. (2000) involved participants who were randomly assigned in pairs as narrators or listeners. Participants engaged in an interaction where narrators told a detailed story with a dramatic ending. The listeners were split into four conditions, one of which, relevant to this issue, was to listen to narratives while simultaneously participating in a distracting task. It was found that listeners in this condition provided significantly fewer instances of listener feedback than participants in the other conditions who were not being distracted. From the narrators’ perspective, reduced listener feedback was experienced as disruptive, and narrators therefore produced less coherent and satisfying stories. Complementary evidence shows that removal of listener feedback results in speakers having to use more words to convey a message (Krauss & Weinheimer, 1966). These data demonstrate that speakers rely on listener feedback for collaborative and successful conversation.

### The Relationship Between Eye Contact and Listener Feedback

Visual contact (direct eye contact or at least visual contact with the interlocutor’s face) plays an important role in the perception and production of listener feedback (Vranjes et al., 2018). From the speaker’s perspective, maintenance of visual contact with the listener allows for the detection of nonverbal listener feedback which signals the listener’s attention and comprehension. And, vice versa, eye contact is crucial for the listener, since effective listener feedback requires the listener to detect, and respond to, gaze cues that indicate the speaker welcomes feedback (Fujie et al., 2005; Ward & Bayyari, 2007). Bavelas et al. (2002) investigated the link between eye contact and listener feedback. The study involved 18 unacquainted psychology students who were paired and given the role of speaker or listener. The speaker had to tell a close-call story while the listeners’ instances of feedback were recorded along with the onsets and offsets of mutual gaze, i.e. periods when participants were simultaneously looking at one another. Findings revealed the listener tended to produce feedback when the speaker looked at them, and the speaker tended to look away soon after the listener gave feedback. Another study (Sandgren et al., 2012) found that even when listeners were visually distracted by a challenging task, their gaze intermittently returned to the speaker’s face for the purpose of asking questions and verbal listener feedback. This enabled the conversational partners to collaborate and succeed on the task. Together, speakers and listeners created and used mutual gaze to coordinate their communication.

Not only do moments of mutual gaze regulate the timing and occurrence of listener feedback, they also affect whether the listener uses visual/nonverbal signals like head nods or auditory/verbal signals, like ‘mhm’ (Erickson & Shultz, 1982). Studies show that listeners use more nonverbal signals than verbal when they are engaged in mutual gaze with the speaker (Eberhard & Nicholson, 2010; Truong et al., 2011). In summary, gaze at a conversational partner is highly relevant for listener feedback, which is used to shape the continuation of the conversation.

### Listener Feedback in ASD

While no previous study has explored the use of all types of listener feedback generally (i.e., verbal and nonverbal signals) in ASD, there is evidence that individuals with ASD provide *certain* nonverbal listener signals less frequently than NT peers, specifically head nods and shakes. For example, a study involving six-minute conversations with a partner about schools, friends, and holidays, found participants with ASD were less likely to nod while listening to their partners (Capps et al., 1998). Complementary research reported an absence of head nods/shakes from 12 ASD adolescent participants compared to 12 NT peers when an interviewer was talking to them (Garcia-Pérez et al., 2007). Although these studies did not set out to examine listener feedback specifically, as they looked at conversation behavior in general, they do indicate that individuals with ASD provide less nonverbal listener feedback, which may be detrimental to conversational clarity and lead to increased communicative breakdown. The current study builds on this work by examining the use of *all* listener feedback, including verbal signals (e.g., backchannels) and including other nonverbal signals (e.g., responsive facial expressions) that have not previously been explored.

As outlined earlier, maintaining facial gaze with the speaker is important to cue the onset of listener feedback in face-to-face conversations. When a speaker glances at the listener, this can signal a moment when the speaker is checking that her message is being attended to and understood. In these moments, the listener should use feedback to indicate attention and comprehension (either verbally or nonverbally). If a listener is not attending to the speaker’s face, s/he should miss these cues. This aspect of listener feedback should be challenging for individuals with ASD since there is lots of evidence that individuals with ASD have atypical social gaze to other people’s faces and eyes compared to NT individuals (Falck‐Ytter et al., 2013; Guillon et al., 2014). Thus, reduced face-directed gaze in this population could have a significant negative impact on their listener feedback, in that individuals with ASD may simply miss moments when they should provide feedback and will therefore provide less feedback than NT peers. Further, a lack of eye contact could result in fewer *nonverbal* listener signals in ASD, specifically, since nonverbal signals are only appropriate when the speaker is looking at the listener (Eberhard & Nicholson, 2010; Truong et al., 2011). A listener who is not visually attending to the speaker’s face should not be able to tell when the speaker is looking at them and will therefore not recognize that a nonverbal (vs. verbal) signal of attention is warranted. In this case, individuals with ASD should show significantly fewer listener responses, generally, since they are missing moments for providing them. However, there is increasing evidence that the context and methodology of social gaze measurements may significantly influence the amount of time individuals with ASD explore others’ faces, with some studies finding no difference between the amount of social gaze between ASD and NT individuals (Falck-Ytter, 2015; Grossman et al., 2019; for review Papagiannopoulou et al., 2014), Therefore, it is possible that when individuals with ASD play the role of the listener, they *do* maintain enough visual contact with their interlocutor in order to detect when the speaker is looking at them. However, they may still produce less listener feedback than NT peers during these moments, because they do not recognize that the speaker is seeking feedback. Struggles with interpreting others’ nonverbal behavior are well documented in ASD (e.g., Olney, 2000). In this case, while NT individuals should produce more listener feedback during moments of mutual gaze, individuals with ASD should produce a similar amount of feedback whether they are sharing gaze with the speaker or not.

### Current Study

Despite the fact that listener feedback and its relationship to social gaze are crucial for conversation, there has been no research to date that has specifically examined how individuals with ASD use listener feedback in conversation. The current study addresses this gap in the literature by examining the frequency of *all* listener feedback (nonverbal and verbal signals, including backchanneling) by older children and adolescents with and without ASD, and explores how moments of mutual gaze impact the frequency of feedback.

In face-to-face interactions between participants and a research assistant (RA) we hypothesize that adolescents with ASD will show reduced frequency of verbal and nonverbal listener feedback compared to NT peers and that this difference will be more apparent when the listener and speaker are looking at one another (mutual gaze). We also hypothesize that higher levels of social communication difficulties (measured by the Social Communication Questionnaire [SCQ]) scores will be related to decreased listener feedback frequency across both diagnostic groups.

## Methods

### Participants

Forty-six ASD and NT participants aged 10–17 years were recruited through local schools, advertisements placed in local magazines, newspapers, the Internet, advocacy groups for families of children with ASD, and word of mouth. The Institutional Review Board of Emerson College Boston, Massachusetts approved this study. The study also received ethical approval from the University of Bath Ethics Committee. We obtained written informed consent from each participant’s parent or guardian as well as the participant themselves if they were over the age of 12. We also obtained verbal assent from participants prior to each research task. Participants were compensated for their time with Amazon gift cards.

We confirmed ASD diagnosis for the ASD sample via the Autism Diagnostic Observation Schedule, 2nd edition (ADOS-2, Lord et al., 2012) by administrators who achieved research reliability with a certified trainer. NT controls were not included if they had a sibling with ASD. All participants completed the Core Language Subtests of the Clinical Evaluation of Language Fundamentals, 5th Edition (CELF-5; Wiig et al., 2013) to assess their language and communication skills in a variety of contexts. We used the Kaufman Brief Intelligence Test, 2nd Edition (K-BIT-2; Kaufman, 2004) to assess IQ. For inclusion in the study, participants had to have a minimum score of 85 on both the CELF and K-BIT determining that all ASD participants had language and cognitive abilities within the normal range. Caregivers for ASD and NT participants completed the SCQ (Rutter et al., 2003), a widely used screen for social communication ability. Children from the NT group were excluded if their scores on the SCQ indicated a possible risk of ASD (scores > 15). Furthermore, participants were only included in the final dataset if their eyes could be successfully tracked for at least 70% of the conversational interaction. Three participants (one ASD and two NT) were excluded due to technical issues with eyetracking. These inclusion and exclusion criteria resulted in a final sample of 20 ASD (16 male, four female) participants and 23 NT participants (14 male, nine female). The mean age in years for the ASD group was 13.8 (SD = 2.01) and for the NT group 13.4 (SD = 2.44). See Table 1 for a descriptive summary of the demographic information across groups.

**Table 1:** Mean scores (SD) and sex for the two groups

	ASD	NT
Age	13.8 (2.01)	13.4 (2.44)
Sex (m, f)	16, 4	14, 9
K-BIT-2	116.2 (18.22)	111.78 (13.64)
CELF-5	109.6 (16.89)	112.87 (16.41)
SCQ	19 (6.77)	2.48 (2.48)

Higher K-BIT score = greater IQ. Higher CELF score = greater language abilities. Higher SCQ score = more social impairments

The two groups did not significantly differ in the ratio of males to females (χ^2^(1, *N* = 43) = 1.86, *p* = 0.17), age (*t*(41) = 0.60, *p* = 0.553), IQ (*t*(41) = 0.91, *p* = 0.370), or language ability (*t*(41) = 0.64, *p* = 0.524). As expected, the two groups differed significantly in their scores for the SCQ (*t*(41) = 10.90, *p* < 0.001). The mean SCQ score for the ASD group was 19 (SD = 6.77) and for the NT group 2.48 (SD = 2.48), showing that the ASD cohort had significantly more social communication impairments.

### Materials

**Fig. 1:**
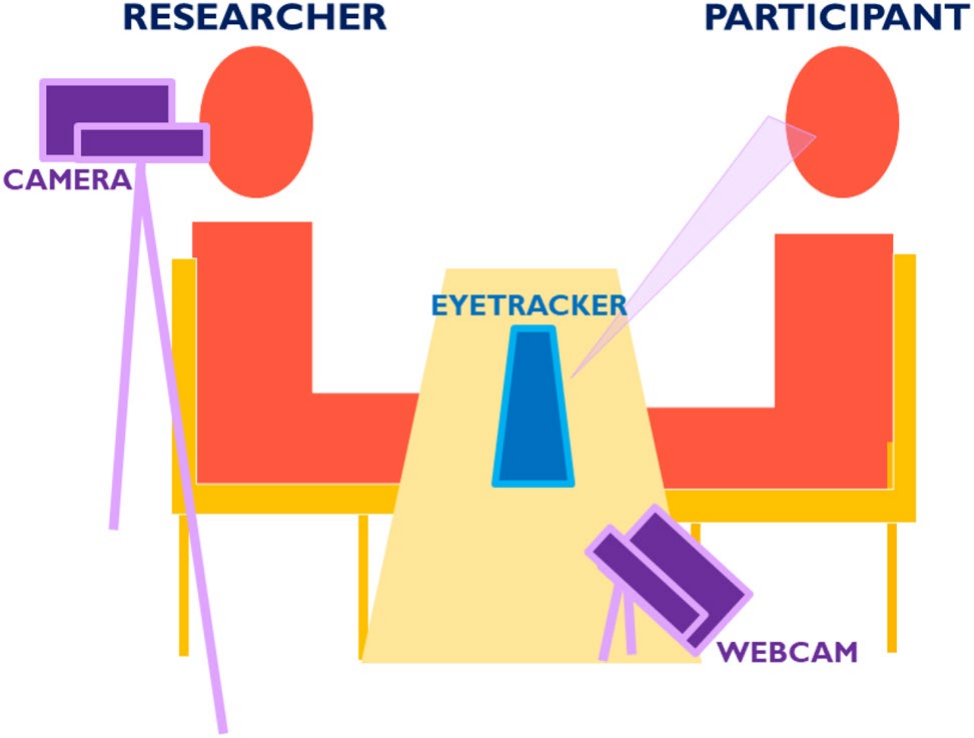
Experiment set-up

We video-recorded the participant’s face and torso using a HDV camera mounted on a tripod and placed three feet behind and to the side of the RA. We simultaneously recorded the RA’s face and torso using a webcam mounted on the table about a foot to the side of the participant and at approximately the same height as their head, so that it could face the RA directly. The eyetracker described below also filmed the RA’s face. See Fig. 1.

Concurrent with video-recording, we used a SensoMotoric Instruments (SMI) RED (RED) eye-tracker to record participant’s eye movements (sampling rate at 100 Hz). We affixed the eye-tracker to a freestanding base and placed it on a small table with two chairs either side. This allowed for adjustment of up/down angle, as well as a small riser to adjust height, as necessary. We used Velcro to attach the eye-tracker to the table to ensure it did not move during the task. Eyetracking was frame-synched with the webcam video of the RA’s face, so that gaze locations could be superimposed onto video of the recording volume.

### Procedure

We utilized a semi-structured face-to-face interaction between participants and a RA. Participants’ listener feedback was recorded in relation to two gaze conditions (during mutual gaze and not during mutual gaze) and different types of listener feedback (verbal and nonverbal). The interactive task was modeled on the Double Interview (Garcia-Winner, 2002) during which an RA and a participant take turns interviewing one another. Participants sat at a small table across from the RA, who faced the participant. See Fig. 1. To begin, when the participants sat down, we completed a five-point eyetracking calibration, aiming for < 1∘ of deviation in either axis. To conduct calibration, we asked participants to look at each one of five dots printed on a laminated sheet of paper (one dot in the center of the sheet and one in each corner), and ensured that the eyetracker’s determination of the point of fixation was within 1∘ of each dot. In order to make sure that calibration values were representative of participant gaze on the RA’s face, the RA sat on a chair during calibration and the sheet of paper was placed directly in front of their face and neck and we adjusted the webcam so that only the sheet was in view. Thus, the shape and location of this sheet defined the spatial extent of the volume within which eye-tracking would be recorded throughout the interview task. We moved the calibration sheet out of the way once calibration was complete so that the RA and participant could communicate with each other freely across the table.

The RA would instruct the participant they were about to have a conversation where, first, the RA would ask questions of the participant that were related to family, holidays, hobbies, and school such as “who are all the people in your family and do you all get along?” Participants were instructed the roles would then be reversed, and that it would be the participant’s job to ask the RA similar questions. Different RAs played the role of interviewer during this task, but they all relied on the same script during the first half of the interview. Consequently, each RA asked the same questions of participants and therefore provided each participant with the same model for the second half of the interview (when the participant would ask questions and listen to the RA’s answers). This ensured that similar conversation topics and context occurred for each interview, thus enabling comparison of performance across groups, and providing a balance between naturalistic conversation and methodological control.

The focus of this investigation was on the second portion of the interview, when it was the participant’s turn to interview the RA. Before starting the second portion of the interview, the participant was shown photographs of the RA with different people and/or in different locations. These photographs were used to help participants generate interview questions (e.g., “It looks like you went to Disneyland. When did you go and who did you go with?”). When showing the participant the photographs the RA would ask them to describe what they saw to check their understanding. Once understanding was confirmed the interview time started and the photographs were removed in order to not impact gaze. The RA was instructed to avoid using prompts once it was the participant's turn to interview them. On rare occasions participants paused for a long period after only asking one or two questions, leading the RA to prompt with “do you have anything else to ask me?” or “do you have any other questions about the photographs?” If the participant said no, the interview ended. If participants did not say no, but were still struggling to create a new question, the photographs were held up for a few seconds over the researcher's face so that gaze was still directed toward the conversation partner while participants looked at the photographs. Neither listener responses nor gaze were coded during these moments, since gaze-to-face was obstructed for both participants.

This interaction task was designed to mimic realistic conversations, in that we did not manipulate the speaker’s gaze or limit the interaction to a given number of seconds. While the RA aimed for each interview to last around 3–4 min this did not always happen because, to ensure the interviews were as natural as possible, conversation was not forced and the RA allowed the interviews to come to a natural end. The participant-led portion of the interview lasted an average of 4.12 min, with a range of 1.93–8.43 min in the ASD group and an average of 3.5 min with a range of 1.18–12.01 min in the NT group. There was a statistically significant difference in mean interview length between ASD and NT groups, *t*(41) = 2.030, *p* = 0.049. To account for this difference, we divided each participants’ listener feedback count by the length of their interview to calculate frequencies of listener feedback per minute.

### Listener Feedback and Gaze Coding

We defined the beginning of the conversational exchange as the moment when the participant asked their first question and the end as the moment when the RA finished providing their final answer. We imported the resulting videos into ELAN software (ELAN, 2018), which was used to code verbal and nonverbal listener feedback from video and audio channels.

To determine what should be coded as listener feedback we consolidated existing relevant literature and found work that included verbal feedback only (Duncan, 1974; Krauss et al., 1977; Lake et al., 2011), nonverbal feedback only (Bertrand et al., 2007; Jonsdottir et al., 2007; Bavelas & Gerwing, 2011), and some that included both (Hess & Johnston, 1988; Morency et al., 2008). In the end, we decided to include both types of listener feedback, so that our analysis would represent a holistic view of listener behavior in ASD. Our final codes included the following: Nonverbal feedback incorporated head shakes, head nods and responsive facial expressions; verbal feedback included backchannels, restatements, clarifications, completions, and laughter. These types of listener feedback have been defined by Yngve (1970), Duncan (1974) and Duncan & Fiske, (1977). As explained in the introduction, backchannels are minimal, short utterances that are non-interruptive (e.g., m-hm). Sentence completions are when the listener completes a sentence that the speaker had already begun but does not continue beyond this brief completion and the speaker continues as if uninterrupted (e.g., S: “I’m not good with spice and accidentally ordered a vindaloo curry which…” L: “was so hot!” S: “…and I had to…”). Brief restatements are similar to completions except the listener immediately restates a few words expressed by the speaker. Clarification requests are also brief, they are the use of a few words to confirm what the speaker said or meant but do not take the speaking turn (e.g., S: “…they’re better at dealing with it …” L: “The stress?” S: “…the stress”). Since this is a preliminary study about listener feedback in ASD, generally, we only analyzed the two larger categories of listener feedback (verbal vs. nonverbal) rather than examining frequencies of different subtypes (e.g., backchannels vs. completions).

Two independent, trained coders identified when listener feedback was produced by the participants during the second half of the interview. During training, coders were taught the definition of listener feedback and they were shown examples of listener feedback in conversations (videos of participants who were excluded from the experimental dataset). The coders then coded listener feedback in five practice interview files. Coders were taught to watch the videos very carefully, and to use assistive tools such as zooming in and reducing the speed, to accurately identify listener feedback and code them appropriately. The first author then went over the practice coding with them to check they were accurate. Any questions the coders had were answered prior to them carrying out any coding used in the analysis reported here.

After undergoing training and practice coding, the two coders independently coded listener feedback for all participant videos. Their coding of listener feedback was checked for reliability by comparing each moment when one coder indicated feedback. If the other coder did not identify feedback in that moment, a third independent, trained coder was called in to watch the same video and to mark moments of listener feedback. Where the third coder agreed with one of the initial coders (either by identifying feedback or by coding no feedback), the majority view was accepted. On the rare occasion that the third coder presented a third alternative (e.g., one coder marked a backchannel when another marked a restatement and a third marked a completion), then the coders watched the moment together, discussed what they saw and why they thought it should be coded one way or the other, and made a final determination by consensus. All three coders were blind to the diagnosis of the participant in each video.

Coders used eyetracking recordings to determine moments of mutual gaze in the video of the RA, which included an overlay of the participant’s scan path as recorded by the eyetracker. We coded “participant gaze” as all the times when the participant’s gaze marker fell within the area of the RA’s face. When the RA in the same video appeared to be looking towards the participant’s face we marked “RA gaze”. Since the video was recorded from the same vantage point as the participant, we were able to determine times of face directed gaze relatively easily. The identification of RA and participant gaze was also checked for reliability. Similar to the procedure for listener feedback coding, when there was a disagreement for gaze coding a third trained coder was brought in. Once reliability was confirmed, when the moments of RA gaze overlapped with the participant gaze we designated them as periods of ‘mutual gaze’. This measure allowed us to compare the number and type of listener feedback that occurred within periods of mutual gaze vs. periods when one or the other interlocutor, or both, were looking away.

### Analysis

To test our hypotheses that NT adolescents would provide more listener feedback than their ASD peers and that group differences would increase during times of mutual gaze, we conducted a 2 (group: ASD, NT) × 2 (condition: mutual gaze, no mutual gaze) repeated-measures ANOVA to analyze the frequency of listener feedback. The dependent variable (DV) was the frequency of listener feedback per minute, as measured by dividing the total number of listener feedback instances per person by the length of their interview in minutes. On the condition that the first ANOVA would show significant group differences, we planned to then conduct a 2 (group: ASD, NT) × 2 (feedback modality: verbal vs. not verbal) repeated-measures ANOVA to see whether differences in frequency of listener feedback between groups was driven by one or other type of feedback (verbal or nonverbal feedback). Finally, to assess the relationship between SCQ scores and listener feedback frequency (all, verbal and nonverbal) we conducted three Spearman’s rank-order correlations on the combined participant group. This non-parametric test was selected as the SCQ scores were not normally distributed, as assessed by a Shapiro–Wilk test (*p* < 0.05).

## Results

For our main analysis, we found a significant main effect of condition, *F*(1, 41) = 6.49, *p* = 0.014, η^2^G = 0.080, with a greater mean frequency of listener feedback in the mutual gaze condition (*M* = 3.13, SD = 2.74), than the no mutual gaze condition (*M* = 1.98, SD = 1.36). There was also a significant main effect of group, *F*(1,41) = 14.00, *p* < 0.001, η^2^G = 0.134, with lower frequency of listener feedback in the ASD group (*M* = 3.4, SD = 2.76) than the NT group (*M* = 6.56, SD = 2.66), regardless of the condition. We did not find a significant interaction between group and condition, *F*(1, 41) = 2.60, *p* = 0.115, η^2^G = 0.033. The descriptive statistics for this can be seen in Table 2.

**Table 2:** Descriptive statistics for frequency of listener feedback per minute

Condition	Diagnosis		Total (n = 43)
	ASD (n = 20)	NT (n = 23)	
Mean frequency gaze (SD)	1.91 (2.20)	4.19 (2.77)	3.13 (2.74)
Mean frequency no gaze (SD)	1.54 (1.11)	2.36 (1.46)	1.98 (1.36)
Mean frequency verbal (SD)	2.08 (1.71)	1.82 (1.69)	1.94 (1.66)
Mean frequency nonverbal (SD)	1.67 (1.69)	5.14 (3.29)	3.53 (3.13)
Overall mean frequency (SD)	1.73 (1.73)	3.28 (2.38)	5.11 (3.10)

The results of the second ANOVA, conducted to determine whether the group difference in listener feedback frequency was specific to verbal or nonverbal listener feedback, showed a significant interaction between the condition (modality of listener feedback—verbal vs. nonverbal) and group (NT vs. ASD), *F*(1,41) = 14.57, *p* < 0.001, η^2^G = 0.154. There was a significant main effect of group, *F*(1,41) = 11.25, *p* = 0.002, η^2^G = 0.118.

There was also a significant main effect of condition, *F*(1,41) = 10.57, *p* = 0.002, η^2^G = 0.117, with a greater frequency of nonverbal listener feedback (*M* = 3.53, SD = 3.13) than verbal listener feedback (*M* = 1.94, and SD = 1.66). The descriptive statistics for this can be seen in Table 2. As a post-hoc measure, we conducted a Tukey HSD test to explore the significant interaction between modality and diagnosis. Beginning with within-group comparisons, the Tukey HSD test revealed a significant difference in modality for the NT group (*p* < 0.0001), reflecting significantly more frequent nonverbal listener feedback as compared to verbal listener feedback. For the ASD group, this difference was not significant (*p* = 0.937). Between-group comparisons showed that the frequency of nonverbal listener feedback amongst NT participants was significantly higher than both nonverbal (*p* < 0.0001) and verbal (*p* < 0.0001) listener feedback amongst ASD participants. The difference between verbal listener feedback was not statistically significant between groups (*p* = 0.980), nor was the difference between *verbal* listener feedback amongst the NT group and *nonverbal* listener feedback in the ASD group (*p* = 0.996).

**Fig. 2:**
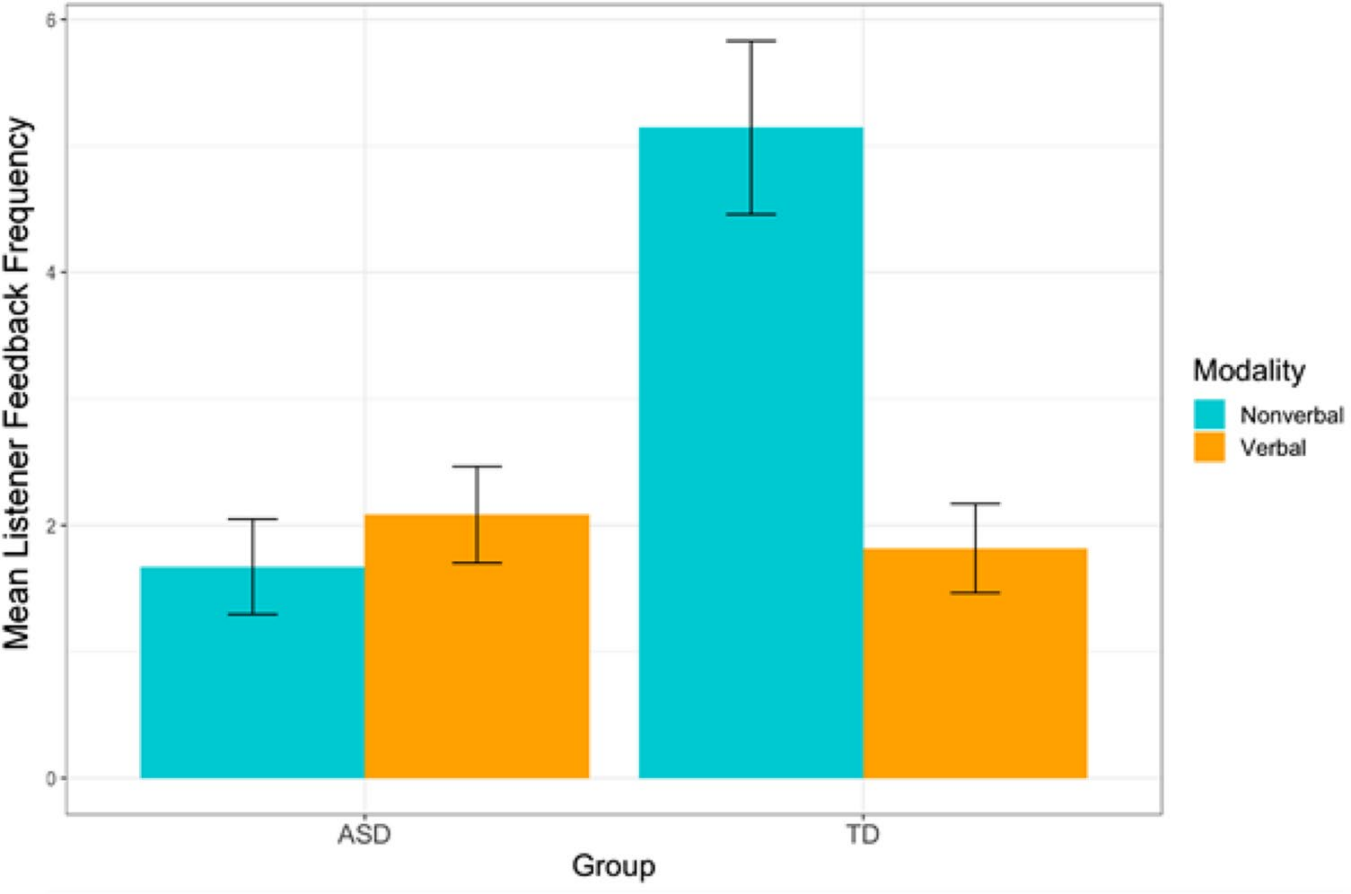
Bar graph with 95% CIs for the mean frequency of verbal vs. nonverbal listener feedback for ASD and NT groups

There was no significant group difference in mutual gaze time (in seconds) across groups (ASD *M* = 85.3 s, SD = 66.09, NT *M* = 74.73 s, SD = 72.78, 95% CI [− 32.50, 53.64], *t* (41) = 0.50, *p* = 0.62). Additionally, there was no significant difference in the proportion of mutual gaze time per interview length across groups (ASD *M* = 0.30, SD = 0.21, NT *M* = 0.32, SD = 0.16, 95% CI [− 0.14, 0.09], *t*(41) = 0.40, *p* = 0.69) (Fig. 2).

**Fig. 3:**
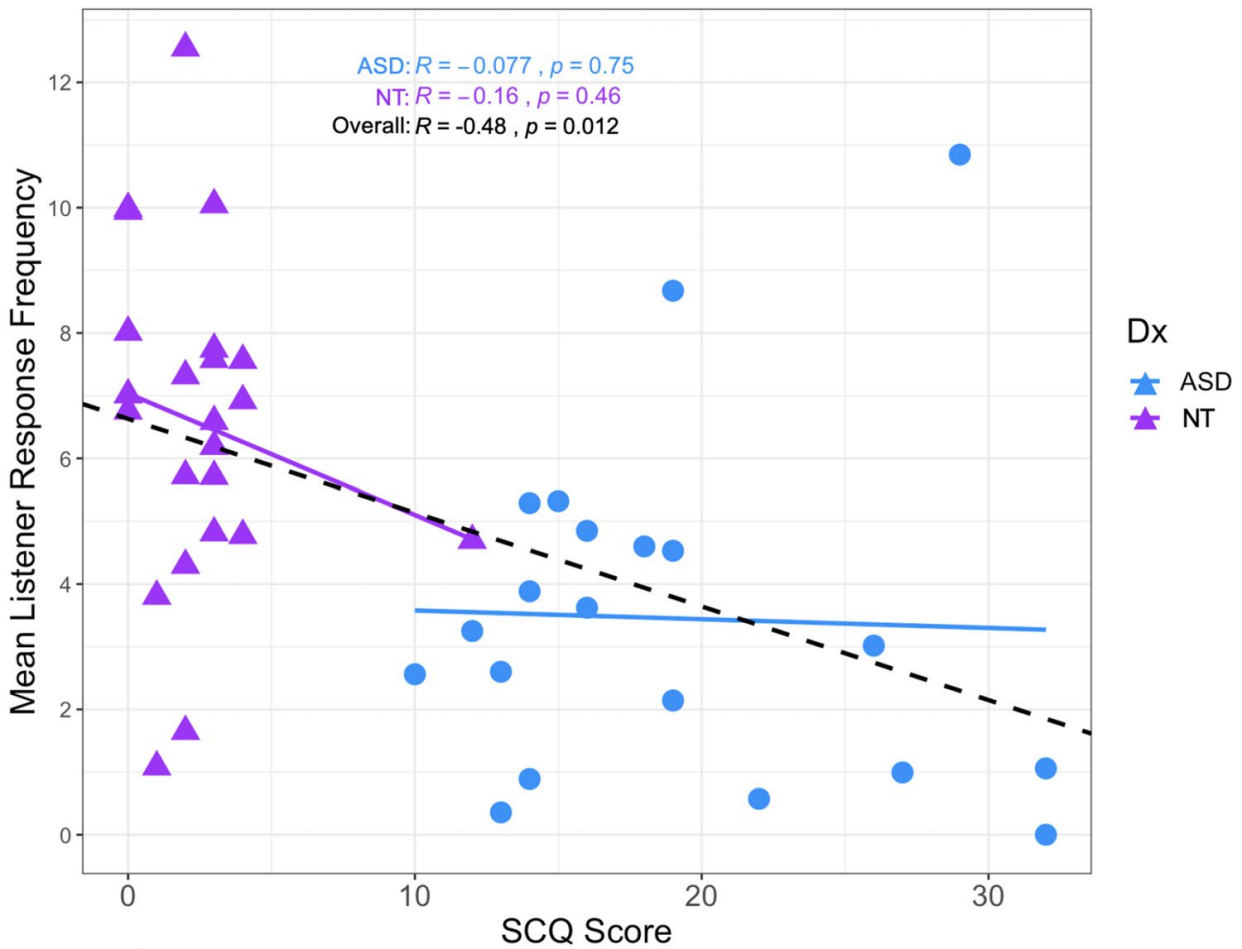
Scatterplot of listener feedback frequency and SCQ scores including lines of best fit for TD and ASD groups

The Spearman’s rank-order correlation found there was a statistically significant, moderate, negative correlation between SCQ scores and frequency of listener feedback, *r*_*s*_(41) = -0.478, *p* = 0.001. That is, as SCQ scores increase, listener feedback frequency decreases, as displayed in Fig. 3. When correlations between SCQ scores and listener feedback frequency were run *within* groups, results were not significant. Further to this, when assessing the relationship between SCQ scores and frequency of just *verbal* listener feedback we found a non-significant, very weak, positive correlation, *r*_*s*_(41) = 0.10, *p* = 0.75. Whereas for SCQ scores and frequency of *nonverbal* listener feedback a statistically significant, moderate, negative correlation, *r*_*s*_(41) = -0.58, *p* < 0.00001 was found.

Finally, given the large range of interview lengths within and between groups, we used Pearson’s correlations to measure the relationship between interview length and listener feedback count^1^ for each group. For the NT group this correlation was significant, showing a strong positive relationship, *r*(41) = 0.92, *p* < 0.0001; for the ASD group, there was a non-significant, weak positive correlation, *r*(41) = 0.29, *p* = 0.066.

## Discussion

The results from this study support our hypothesis that adolescents with ASD provide listener feedback less frequently than their NT peers. Participants with ASD produced less listener feedback, no matter whether they were engaged in mutual gaze or not. This is an important finding, since listener feedback is an integral part of successful communication (Levinson & Enfield, 2006). Relatively infrequent listener feedback could be a contributing factor in why interactions with individuals with ASD are perceived as awkward by others (Brinton & Fujiki, 1989; Marans et al., 2005). As described in the introduction, when listeners produce little or no listener feedback, speakers feel less comfortable in the interaction and have to use more words to convey their message (Bavelas et al., 2000; Marans et al., 2005). Such effects may have occurred in the current study because the participants with ASD gave fewer indications to their interlocutor they were listening or engaged in the interaction. As a result, their conversation partners may have spoken less cohesively and used more words, leading to the significantly longer interview lengths for the ASD group than for the NT group.

In the NT group, as interview length increased so did occurrences of listener feedback. This may suggest that the child’s listener feedback contributed positively to the maintenance of the interaction, i.e., that RAs spoke more when the child indicated engagement by producing listener feedback and/or that children who produce more listener feedback were more interested/motivated in sustaining the interview. However, the relationship could indicate that longer interviews had more listener feedback simply because there were more opportunities for them. In contrast to the NT group, there was not a significant relationship between interview length and listener feedback count for interactions with ASD children. This suggests that factors aside from listener feedback contributed to longer interactions, and it suggests that lower rates of listener behaviors in the ASD group are not dependent on fewer opportunities to produce them.

The non-significant correlation between SCQ scores and listener feedback frequency when run within groups suggests that the overall significant correlation between SCQ and listener feedback frequency was driven by group differences in SCQ scores (significantly higher in the ASD group) and group differences in listener feedback frequency (significantly lower in the ASD group). It is interesting that SCQ scores were significantly negatively correlated with listener feedback for the entire participant group, indicating that social communication ability, rather than diagnostic category, may be foundational to our findings. Further investigation of the relationship between SCQ scores and type of listener feedback showed there was only a significant negative correlation for nonverbal listener feedback. This complements literature that found sensitivity to, and understanding of, nonverbal cues was associated with a lower number of autistic characteristics in a sample of NT undergraduates (Ingersoll, 2010). However, we should interpret these findings carefully, since the relationship between SCQ scores and listener feedback was not found within groups. Within-group correlations show that individuals with ASD with higher SCQ scores did not provide listener feedback significantly less frequently than individuals with ASD with lower SCQ scores. And similarly, NT individuals with higher SCQ scores did not provide listener feedback less often. Therefore, we interpret our results for this hypothesis (SCQ scores will negatively correlate with the frequency of listener feedback) as simply confirming results for our first hypothesis (group differences): Adolescents with ASD – who have significantly higher SCQ scores – provide listener feedback (specifically nonverbal feedback) significantly less often than NT adolescents.

Interestingly, NT individuals produced significantly more nonverbal forms of listener feedback than ASD individuals but there was no significant difference between groups in the frequency of verbal forms of listener feedback. It is possible that ASD individuals, who have difficulty reading the nonverbal cues of others (Olney, 2000), do not recognize nonverbal listener feedback as an important aspect of conversation. They may not acquire nonverbal listener feedback through observation of others or understand why it is relevant and therefore produce nonverbal listener feedback less than NT individuals. For example, studies have shown ASD individuals were less likely to nod or shake their head when listening to conversational partners than NT controls (Capps et al., 1998; Garcia-Pérez et al., 2007).

Another possible explanation for the ASD group providing less nonverbal listener feedback is that they may not realize when such feedback is required or expected. Existing studies show that individuals with ASD find it hard to pick up on verbal and nonverbal cues from the speaker such as the prosodic features (pausing, upward intonation that indicates questioning) and facial expressions (when the speaker might glance toward the listener to ensure they’re paying attention/understanding) which signal the need for listener feedback (Ward & Tsukahara, 2000). Even ASD individuals with average or above average language scores, seem to have reduced ability to produce nonverbal social signals (Garcia-Pérez et al., 2007). In our study, ASD individuals’ intact verbal abilities may have enabled them to produce verbal, but not nonverbal listener feedback. Future research should look specifically to identify moments where the speaker was actively seeking or providing opportunity for listener feedback to determine whether individuals with ASD respond less frequently to those cues or invitations.

Alternatively, one might suspect that ASD individuals produce fewer nonverbal forms of listener feedback because they look at their conversation partner less. This would be consistent with findings of atypicality in eye contact in ASD literature (Guillon et al., 2014). If NT individuals had more time looking at their conversation partner, they would receive more visual cues from the speaker to provide feedback, and then they could accordingly provide more nonverbal feedback. However, other research using similar methods to ours, found ASD participants did not look at their conversation partner any less than NT controls did (Fletcher-Watson et al., 2009; McPartland et al., 2011; Grossman et al., 2019) and higher autistic traits were not associated with reduced looking at the social partner (Vabalas & Freeth, 2016). Our findings support these results as the mutual gaze time in both groups was the same, even when taken as a proportion of each participants’ interview length. This finding further strengthens the noted difference in listener feedback for ASD and NT individuals: even though ASD and NT participants had similar opportunities to see speaker’s prompts for listener feedback, there were significant differences between the amounts of nonverbal feedback produced during moments of mutual gaze for the two groups. Overall, our findings show that adolescents with ASD are sharing gaze with their conversational partner similarly to their NT peers, but are not providing the same level of visual/nonverbal listener feedback during those times of mutual gaze. These data indicate that individuals with ASD may not recognize the need to provide such feedback or cues from speakers seeking feedback, despite having the same level of visual access to their conversation partner as NT individuals.

Our data also confirm our hypothesis that significantly more listener feedback would occur during times of mutual gaze than times of no shared gaze across all participants. This is consistent with findings that show gaze is used by the speaker as a cue to request listener feedback (Sandgren et al., 2012) and studies on NT individuals that found feedback occurs when the speaker looks to the listener for acknowledgement (Bavelas et al., 2002). Moments of mutual gaze also provide opportunity for more listener feedback, because when there is mutual gaze the listener is not restricted to just verbal listener feedback and instead can provide both verbal and nonverbal listener feedback meaningfully (Bertrand et al., 2007). Our hypothesis that individuals with ASD would show less of a difference in frequency of feedback across gaze conditions (during mutual gaze vs. not during mutual gaze) was also supported by our data.

This research can potentially inform interventions to help address the social communication difficulties of individuals with ASD. Interventions targeting the appropriate and effective use of listener feedback could be of critical importance. Interventions aiming to enhance or remediate the conversational performance of individuals with ASD have targeted essential conversational skills like maintaining a topic, topic shift, turn taking, repairing breakdowns and checking for understanding and interruptions (Charlop et al., 2008; Koegel et al., 2014). The results from this study suggest that interventions focusing on nonverbal listener feedback and their relation to gaze, could also prove beneficial.

### Limitations

The results from this study should be interpreted in the light of some limitations. One limitation is that the RA’s listener feedback was not controlled for in the first half of the interview where the participant was the speaker and the RA was the listener. The RA’s listener feedback may have varied for each participant depending on who the RA was, their mood during the testing session, and/or how they related to their conversational partner. The frequency/type of listener feedback produced by the RA in the first half of the interview may have affected the way the child did it in the second half, in that it may have modelled this behavior for the participant. Further, it is known that NT individuals are more influenced by their conversational partner as they imitate their speech, gestures, expressions and posture (Richardson et al., 2008) than individuals with ASD (Hobson & Meyer, 2005). Therefore, not only may participants have been impacted by their partner’s listener feedback, but also participant groups may have been *differently* impacted by their partner’s feedback behaviors.

Another limitation of our findings is that we are only able to speak to the frequency of feedback, but not its quality or timing. These features are important as they impact the quality of the interaction. Listener feedback that is delayed or mistimed negatively affects an interaction (Ward et al., 2007). And inappropriate responses – for example, laughter that occurs after the speaker says something serious – will also obviously affect the interaction. Our coding system did not account for this. We interpret our results as a preliminary investigation into listener responsiveness in ASD, and we encourage future studies to follow up on our findings, by examining other aspects of listener feedback, like quality and timing.

One strength of the present study is the use of an ecologically valid, natural dyadic face-to-face interaction to measure listener feedback, rather than a very structured interaction (for instance, one where the RA produced speech from a script). While this provides a more spontaneous interaction, there are limitations in this method, in that we did not ensure consistency from interview to interview (as described above). Different participants asked the RA different questions, and the RAs then provided different answers. This variation likely impacted opportunities for listener responsiveness in each interview. Conversely, the fact that we recorded listener feedback during a semi-structured interview, rather than an unstructured conversation, makes it difficult to determine whether responsiveness would generalize to more spontaneous interactions. Additionally, interactions were with unknown adults rather than with a familiar person, so again, it is difficult to know whether listener behaviors in the current study would generalize to interactions with friends/family. While it would be elucidating to measure responsiveness in truly natural/spontaneous conversations with familiar people, such a study significantly reduces the ability to control topic and format across participants.

Finally, for a few participants there was a moment during the interview during which gaze to the face was not captured. This moment happened when photographs were held up, covering the RA’s face, for participants who were struggling to create interview questions. Future research in this area should avoid the inclusion of any extraneous visual stimuli that might disrupt or obstruct gaze from the region of interest (the interlocutor's face).

## Conclusions

Our study aimed to determine whether adolescents with ASD produce less listener feedback than their NT peers, particularly during times of mutual gaze. Results show that adolescents with ASD provide listener feedback less frequently than NT peers and in particular use fewer nonverbal forms of listener feedback. The findings of the present study may contribute to larger social skills interventions for education and therapy for those with ASD such as self-management, a motivational intervention that helps those with ASD become more aware and monitor their own behavior (Koegel & Koegel, 2006). Interventions could focus on increasing awareness of speaker cues for feedback such as gaze as well as teaching the communicative purpose of listener feedback, particularly nonverbal forms, which could have a positive impact on how individuals with ASD are perceived during conversation.

## Footnotes

1 It is important to underscore that all other analyses used listener feedback *frequency* (the count of listener feedback divided by the length of the interview) as the numerical representation of listener feedback. Thus, our feedback measure always accounts for interview length. Only for this analysis—in which we correlate the number of listener feedback behaviors with interview length—do we use raw feedback counts.

## References

[CR2] Arie M, Tartaro A, Cassell J (2008). Conversational turn-taking in children with autism: Deconstructing reciprocity into specific turn-taking behaviors.

[CR3] Bavelas JB, Coates L, Johnson T (2000). Listeners as co-narrators. Journal of Personality and Social Psychology.

[CR4] Bavelas JB, Coates L, Johnson T (2002). Listener responses as a collaborative process: The role of gaze. Journal of Communication.

[CR5] Bavelas JB, Gerwing J (2011). The listener as addressee in face-to-face dialogue. International Journal of Listening.

[CR6] Bertrand, R., Ferré, G., Blache, P., Espesser, R., & Rauzy, S. (2007). Backchannels revisited from a multimodal perspective. *Auditory-visual Speech Processing*, 1–5.

[CR7] Brinton B, Fujiki M (1989). Conversational management with language-impaired children: Pragmatic assessment and intervention.

[CR8] Capps L, Kehres J, Sigman M (1998). Conversational abilities among children with autism and children with developmental delays. Autism.

[CR9] Carpenter, M., & Tomasello, M. (2000). Joint attention, cultural learning, and language acquisition: Implications for children with autism.

[CR10] Charlop MH, Gilmore L, Chang GT (2008). Using video modeling to increase variation in the conversation of children with autism. Journal of Special Education Technology.

[CR12] Duncan S (1974). On the structure of speaker–auditor interaction during speaking turns. Language in Society.

[CR13] Duncan S, Fiske PW (1977). Face-to-face interaction: Research, methods, and theory.

[CR14] Eales MJ (1993). Pragmatic impairments in adults with childhood diagnoses of autism or developmental receptive language disorder. Journal of autism and developmental disorders.

[CR15] Eberhard, K., & Nicholson, H. (2010). Coordination of understanding in face-to-face narrative dialogue. *Proceedings of the Annual Meeting of the Cognitive Science Society*, *32*.

[CR16] ELAN (Version 5.2) [Computer software]. (2018). Nijmegen: Max Planck Institute for Psycholinguistics. https://tla.mpi.nl/tools/tla-tools/elan/

[CR17] Erickson F, Shultz JJ (1982). The counselor as gatekeeper: Social interaction in interviews.

[CR18] Falck-Ytter T (2015). Gaze performance during face-to-face communication: A live eye tracking study of typical children and children with autism. Research in Autism Spectrum Disorders.

[CR19] Falck-Ytter T, Bölte S, Gredebäck G (2013). Eye tracking in early autism research. Journal of Neurodevelopmental Disorders.

[CR20] Fletcher-Watson S, Leekam SR, Benson V, Frank MC, Findlay JM (2009). Eye-movements reveal attention to social information in autism spectrum disorder. Neuropsychologia.

[CR21] Fujie, S., Fukushima, K., & Kobayashi, T. (2005, September 4–8). *Back-channel feedback generation using linguistic and nonlinguistic information and its application to spoken dialogue system*. Paper presented at the Ninth European Conference on Speech Communication and Technology, Lisbon.

[CR22] García-Pérez RM, Lee A, Hobson RP (2007). On intersubjective engagement in autism: A controlled study of nonverbal aspects of conversation. Journal of Autism and Developmental Disorders.

[CR23] Garcia-Winner M (2002). Assessment of social skills for students with Asperger syndrome and high-functioning autism. Assessment for Effective Intervention.

[CR24] Goodwin C (1986). Between and within: Alternative sequential treatments of continuers and assessments. Human Studies.

[CR25] Grossman RB, Zane E, Mertens J, Mitchell T (2019). facetime vs Screentime: Gaze patterns to Live and Video Social Stimuli in Adolescents with ASD. Scientific reports.

[CR26] Guillon Q, Hadjikhani N, Baduel S, Rogé B (2014). Visual social attention in autism spectrum disorder: Insights from eye tracking studies. Neuroscience & Biobehavioral Reviews.

[CR27] Hess LJ, Johnston JR (1988). Acquisition of back channel listener responses to adequate messages. Discourse Processes.

[CR28] Hobson RP, Meyer JA (2005). Foundations for self and other: A study in autism. Developmental Science.

[CR29] Ingersoll B (2010). Broader autism phenotype and nonverbal sensitivity: Evidence for an association in the general population. Journal of Autism and Developmental Disorders.

[CR30] Jones CD, Schwartz IS (2009). When asking questions is not enough: An observational study of social communication differences in high functioning children with autism. Journal of Autism and Developmental Disorders.

[CR31] Jonsdottir GR, Gratch J, Fast E, Thórisson KR, Pelachaud C, Martin JC, André E, Chollet G, Karpouzis K, Pelé D (2007). Fluid semantic back-channel feedback in dialogue: Challenges and progress. International Workshop on Intelligent Virtual Agents.

[CR32] Kaufman, A. S. (2004). *KBIT-2: Kaufman Brief Intelligence Test. 2nd.* Minnesota: American Guidance Service.

[CR33] Klinger LG, Williams AMIE (2009). Cognitive-behavioral interventions for students with autism spectrum disorders.

[CR34] Koegel L, Park M, Koegel R (2014). Using self-management to improve the reciprocal social conversation of children with autism spectrum disorder. Journal of Autism and Developmental Disorders.

[CR35] Koegel RL, Koegel LK (2006). Pivotal response treatments for autism: Communication, social, & academic development.

[CR36] Koiso H, Horiuchi Y, Tutiya S, Ichikawa A, Den Y (1995). An analysis of turn-taking and backchannels based on prosodic and syntactic features in Japanese map task dialogs. Language and Speech.

[CR37] Krauss RM, Garlock CM, Bricker PD, McMahon LE (1977). The role of audible and visible back-channel responses in interpersonal communication. Journal of Personality and Social Psychology.

[CR38] Krauss RM, Weinheimer S (1966). Concurrent feedback, confirmation, and the encoding of referents in verbal communication. Journal of Personality and Social Psychology.

[CR39] Lake JK, Humphreys KR, Cardy S (2011). Listener vs. speaker-oriented aspects of speech: Studying the disfluencies of individuals with autism spectrum disorders. Psychonomic Bulletin & Review.

[CR40] Levinson SC, Enfield NJ (2006). Roots of human sociality: Culture, cognition and human interaction.

[CR41] Lord C, Rutter M, DiLavore PC, Risi S, Gotham K, Bishop S (2012). Autism diagnostic observation schedule: ADOS.

[CR42] Marans WD, Rubin E, Laurent A (2005). Addressing social communication skills in individuals with high-functioning autism and asperger syndrome: Critical priorities in educational programming. Handbook of Autism and Pervasive Developmental Disorders.

[CR43] McPartland JC, Webb SJ, Keehn B, Dawson G (2011). Patterns of visual attention to faces and objects in autism spectrum disorder. Journal of Autism and Developmental Disorders.

[CR61] Morency, L. P., de Kok, I., & Gratch, J. (2008). Predicting listener backchannels: A probabilistic multimodal approach. In H. Prendinger, J. Lester & M. Ishizuka (Eds.), *International workshop on Iitelligent virtual agents* (pp. 176–190). Berlin: Springer.

[CR60] Mueller, F. E. (1996). Affiliating and disaffiliating with continuers: Prosodic. Aspects of Recipiency. In E. Cooper-Kuhlen & M. Setting (Eds.), *Prosody in conversation: International Studies.* (pp. 113–176). Cambridge: Cambridge University Press.

[CR44] Olney MF (2000). Working with autism and other social-communication disorders. Journal of Rehabilitation.

[CR45] Papagiannopoulou EA, Chitty KM, Hermens DF, Hickie IB, Lagopoulos J (2014). A systematic review and meta-analysis of eye-tracking studies in children with autism spectrum disorders. Social Neuroscience.

[CR46] Richardson, D., Dale, R., & Shockley, K. (2008). Synchrony and swing in conversation: Coordination, temporal dynamics, and communication. *Embodied Communication in Humans and Machines*, 75–94.

[CR47] Rutter M, Bailey A, Lord C (2003). SCQ. *The Social Communication Questionnaire*.

[CR48] Sandgren O, Andersson R, Weijer JVD, Hansson K, Sahlén B (2012). Timing of gazes in child dialogues: a time-course analysis of requests and back channelling in referential communication. International Journal of Language & Communication Disorders.

[CR49] Schröder M, Heylen D, Poggi I, Hoffmann R, Mixdorff H (2006). Perception of non-verbal emotional listener feedback. Speech Prosody.

[CR50] Tolins J, Tree JEF (2014). Addressee backchannels steer narrative development. Journal of Pragmatics.

[CR51] Truong, K.P., Poppe, R., Kok, I., Heylen, D. (2011, August 27–31). *A multimodal analysis of vocal and visual backchannels in spontaneous dialogs.* Paper presented at the Twelfth Annual Conference of the International Speech Communication Association, Florence.

[CR52] Vabalas A, Freeth M (2016). Brief report: Patterns of eye movements in face to face conversation are associated with autistic traits: Evidence from a student sample. Journal of Autism and Developmental Disorders.

[CR54] Vranjes J, Brône G, Feyaerts K (2018). Dual feedback in interpreter-mediated interactions: On the role of gaze in the production of listener responses. Journal of Pragmatics.

[CR55] Ward N, Bayyari YA (2007). A prosodic feature that invites back-channels in Egyptian Arabic. Amsterdam Studies in the Theory and History of Linguistic Science Series.

[CR56] Ward NG, Escalante R, Al Bayyari Y, Solorio T (2007). Learning to show you're listening. Computer Assisted Language Learning.

[CR57] Ward N, Tsukahara W (2000). Prosodic features which cue back-channel responses in English and Japanese. Journal of Pragmatics.

[CR58] Wiig EH, Secord WA, Semel E (2013). Clinical evaluation of language fundamentals: CELF-5.

[CR59] Yngve VH (1970). On getting a word in edgewise: Papers from the sixth regional meeting Chicago Linguistic Society.

